# Supplementary material for: Spartin-mediated lipid transfer facilitates lipid droplet turnover
Source: bioRxiv. 2023 Nov 29:2023.11.29.569220. Preprint. [Version 1] doi: 10.1101/2023.11.29.569220 (PMC10705495; doi:10.1101/2023.11.29.569220)
Supplement: 1 [file NIHPP2023.11.29.569220V1-supplement-1.pdf]

bioRxiv preprint doi: https://doi.org/10.1101/2023.11.29.569220; this version posted November 29, 2023. The copyright holder for this preprint (which was not certified by peer review) is the author/funder, who has granted bioRxiv a license to display the preprint in perpetuity. It is made available under aCC-BY 4.0 International license.

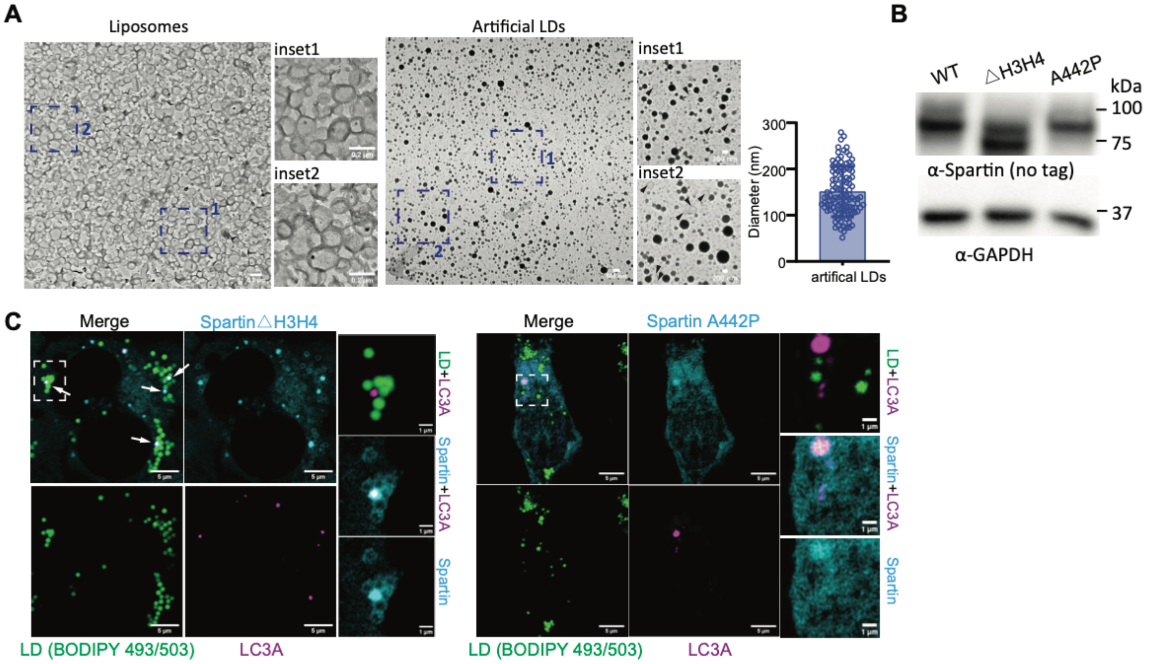

Figure S1. Examination of artificial LDs by EM and characterization of spartin mutants. **A**: Positive staining TEM images of liposomes and artificial LDs (used in Fig1H and Fig2F). Artificial LDs enriched with unsaturated TAG (average diameter=150nm, n>100) are stained by osmium tetroxide as electron-dense black dots. In contrast, liposomes are delineated by lower contrast grey membranes, and their interior appears white. In the insets for the lipid droplet preparations, arrows indicate liposome contaminants. Imaging was carried out using a T12 microscope with a magnification of 2700x at 120kV as in (14). **B**: Western blot shows successful generation of stable cell lines (used in Fig3E) expressing untagged Spartin constructs in KO background. **C**: Fluorescence microscopy shows Spartin$_{\triangle H3H4}$ maintains the ability to tether LD to LC3A-autophagosomes, but Spartin A442P mutant fails to tether as A442P does not localize to LDs anymore (Fig 3G). Stable cells expressing DsRed-Spartin △H3H4 (n=11) and DsRed-Spartin A442P construct (n=9) were transiently transfected with BFP-LC3A and followed by 0.1 mM OA treatment for 18-hr; OA was removed 3hrs before live cell imaging.

bioRxiv preprint doi: https://doi.org/10.1101/2023.11.29.569220; this version posted November 29, 2023. The copyright holder for this preprint (which was not certified by peer review) is the author/funder, who has granted bioRxiv a license to display the preprint in perpetuity. It is made available under aCC-BY 4.0 International license.

Table S1

3xFLAG-*Hs*Spartin FL, pCMV10	F: ctgttccaggggcccccttgcggccgccATGGAACAAGAGCCACA
	R: gtcacagggatgccacccgggatccTCACTTGTCCTTTTTCTTCGCC
3xFLAG-DsRed-*Hs*Spartin FL, pCMV10	F1: ctgttccaggggcccccttgcggccgccATGGACAACACCGAGGACGTCATC
	R1: tgccacttccCTGGGAGCCGGAGTGGCG
	F2: cggctcccagGGAAGTGGCAGTGGTAGTG
	R2: gtcacagggatgccacccgggatccTCACTTGTCCTTTTTCTTCGCC
DsRed-*Hs*Spartin constructs, pLVX	F: gatctcgagctcaagcttcgaattcATGGACAACACCGAGGACG
	R: agaattatctagagtcgcgggatccTCACTTGTCCTTTTTCTTC
3xFLAG-DsRed-*Hs*Spartin A442P, pCMV10	F: GGTCAAAGGTccgGAGATTACCGG
	R: AAACCCCAGCTAACCCAG
3xFLAG-DsRed-*Hs*Spartin △ H3H4, pCMV10	F: GTCGATAACAGCCAACGTGAGAAC
	R: CCCGTCCAGCGGGC
3xFLAG-DsRed-*Hs*Spartin △ MIT, pCMV10	F: AAATTGTACCCGGAGTTC
	R: ATTCTGTGGCTCTTGTTC
3xFLAG-DsRed-*Hs*Spartin △ PPAY, pCMV10	F: GGTCACTATACCGTCAGCTATGGT
	R: CTCCGCCGGACAGCTT
3xFLAG-*Ce*Spartin FL, pCMV10	F: ctgttccaggggcccccttgcggccgccATGTTCTCGATATTTATGAGAATTCC
	R: gtcacagggatgccacccgggatccTCAGACAGCCTTCTTGAGC
*Ce*Spartin 95-240-6HIS, pET29	F: ctttaagaaggagatatacatatgAAAATCAAGGAAGCTGAAG
	R: agtggtggtggtggtggtgctcgagAGCTTGTTCTTTGAAATCC
3xFLAG-*Ce*Spartin 121-476, pCMV10	F: GAAGCTGACTTGATTTATTTTTTGCCGGAAG
	R: GGCGGCCGCAAGG
His-MBP-TEV-*Ce*Spartin FL, pET28	F: cctgtatttccagggagaaggatccTTCTCGATATTTATGAGAATTCCTG
	R: tggtggtggtgctcgagtgcggccgcTCAGACAGCCTTCTTGAG
3xFLAG-*Ct*Spartin 7-480, pCMV10	F: ctgttccaggggcccccttgcggccgccGACCCCAAGCTCCTCTAC
	R: gtcacagggatgccacccgggatccTCAGTGGTGGTGGTGGTGGTG
3xFLAG-*Ct*Spartin 7-245, pCMV10	F: GGTGGCGGTGGTAGTG
	R: GAGGGTGATTTCGACGGGG
3xFLAG-*Ct*Spartin △H1H2, pCMV10	F: GGCGGGCATCCTAAGG
	R: GGATATGGGTGCGATGTTGATCTTG
3xFLAG-*Ct*Spartin △H3H4, pCMV10	F: GGTGGCGGTGGTAGTG
	R: TGGCTTATAAGTAGGCAGTGGC
3xFLAG-*Ct*Spartin 7-480 A290P, pCMV10	F: CATCGCCGGCccaGAAATG
	R: ATCAGGCGCGACGCG
3xFLAG-*Ct*Spartin 7-480 PreScission site, pCMV10	F: ttccaggggcccGATTTTAAGCCTGGTGAGGGCTATG
	R: cagaacttcaagAGCGGGGTTATACGGCG
His-MBP-TEV-*Ct*Spartin 7-480, pET28	F: cctgtatttccagggagaaggatccGACCCCAAGCTCCTCTAC
	R: tggtggtggtgctcgagtgcggccgcTCATCCAACAATGACGCTC
3XFLAG-BFP-LC3A, pCMV10	F1: ctgttccaggggcccccttgcggccgccATGAGCGAGCTGATTAAGGA
	R1: ctgagggcatACTTCCACTACCACTTCC
	F2: tagtggaagtATGCCCTCAGACCGGCCT
	R2: gtcacagggatgccacccgggatccCTAGCCGAAGGTTTCCTGGG
mCherry-GFP-GPAT4, CMV-C1 backbone	F1: ttagtgaaccgtcagatccgctagcGCCACCATGGTGAGCAAG
	R1: taccacttccACTACCACTGCCACTTCCcttgtacagctcgtccat
	F2: cagtggtagtGGAAGTGGTAGTGGAAGTATGGtgagcaagggcg
	R2: tgagtccggaCTTGTACAGCTCGTCCATG
	F3: gctgtacaagTCCGGACTCAGATCTCG

bioRxiv preprint doi: https://doi.org/10.1101/2023.11.29.569220; this version posted November 29, 2023. The copyright holder for this preprint (which was not certified by peer review) is the author/funder, who has granted bioRxiv a license to display the preprint in perpetuity. It is made available under aCC-BY 4.0 International license.

	R3: tcagttatctagatccggtggatccTTAGAACTCCTTAAACCTCCC

Table S2

Data set	CeSpartin FD-Corrected	CtSpartinL WT	CtSpartinL A290P
HDX reaction details	%D_2O=70.7%<br>$pH_{(read)}$=7.5<br>Temp=18ºC	%D_2O=84.9%<br>$pH_{(read)}$=7.5<br>Temp=18ºC	%D_2O=84.9%<br>$pH_{(read)}$=7.5<br>Temp=18ºC
HDX time course (seconds)	3s,30s,300s,3000s	0.3s (3s on ice), 3s,30s,300s	0.3s (3s on ice), 3s,30s,300s
HDX controls	Maximally deuterated sample	Maximally deuterated sample	Maximally deuterated sample
Back-exchange	33.4% ± 16.0%	33.3% ± 13.9%	30.8% ± 13.6%
Number of peptides	180	110	110
Sequence coverage	94.4%	90.3%	90.3%
Average peptide length/redundancy	Length= 14.8<br>Redundancy= 5.6	Length= 17.5<br>Redundancy= 3.8	Length= 17.5<br>Redundancy= 3.8
Replicates	3	3	3
Repeatability	Average StDev=1.3%	Average StDev=1.1%	Average StDev=1.1%
Significant differences in HDX	>4.5% and >0.45 Da and unpaired t-test ≤0.01	>4.5% and >0.45 Da and unpaired t-test ≤0.01	>4.5% and >0.45 Da and unpaired t-test ≤0.01
